# Preventing male suicide through a psychosocial intervention that provides psychological support and tackles financial difficulties: a mixed method evaluation

**DOI:** 10.1186/s12888-022-03973-5

**Published:** 2022-05-13

**Authors:** Joni Jackson, Michelle Farr, Kate Birnie, Philippa Davies, Loubaba Mamluk, Marina O’Brien, Jez Spencer, Rebecca Morgan, Christian Costello, John Smith, Jonathan Banks, Maria Theresa Redaniel

**Affiliations:** 1 The National Institute for Health and Care Research Applied Research Collaboration West (NIHR ARC West) at University Hospitals Bristol and Weston NHS Foundation Trust, Bristol Medical School, University of Bristol, 9th floor Whitefriars, Lewins Mead, Bristol, BS1 2NT UK; 2 Population Health Sciences, Bristol Medical School, University of Bristol, Bristol, UK; 3 Second Step, Bristol, UK

**Keywords:** Evaluation, Intervention, Mixed-methods, Depression, Suicide, Financial advice

## Abstract

**Background:**

To help resolve high suicide rates in Bristol, North Somerset and South Gloucestershire, the charity Second Step was commissioned to roll-out the Hope service offering a psychosocial intervention for men, supporting them through acute distress and addressing financial difficulties. This study evaluated the impact of the Hope service on men at risk of suicide experiencing financial and other difficulties.

**Methods:**

Mixed methods study using: (i) a prospective cohort study design to compare depression, suicidal ideation and financial self-efficacy scores of men aged 30–64, referred to the service between October 2018 and July 2020, at baseline and 6 months follow-up and between low and moderate to high-intensity service users; and (ii) a qualitative interview study to evaluate the acceptability and impact of the Hope service to Hope service users.

**Results:**

There was a 49% reduction in depression score (mean reduction − 10.0, 95% CI − 11.7 to − 8.3) and in the proportion of service users with suicidal ideation (percent reduction − 52.5, 95% CI − 64.1% to − 40.9%) at 6 months follow-up compared to baseline. Financial self-efficacy scores increased by 26% (mean increase 2.9, 95% CI 1.8 to 3.9). Qualitative accounts illustrated how ‘Hope saved my life’ for several men interviewed; most respondents described being able to move forward and tackle challenges with more confidence following the Hope intervention. Professional advice to tackle financial and other difficulties such as housing helped to relieve anxiety and stress and enable practical issues to be resolved.

**Conclusions:**

The Hope service offered practical and emotional support to men who have experienced suicidal feelings, redundancy, homelessness and poverty and occupies an important space between mental health and social care provision. Hope demonstrates the value of an intervention which cuts across traditional boundaries between psychiatric care and social advice agencies to provide, what is, in effect, an integrated care service.

**Supplementary Information:**

The online version contains supplementary material available at 10.1186/s12888-022-03973-5.

## Background

A third of all deaths by suicide are amongst middle-aged men (40–50 years) [1] and in Bristol the rate of suicide deaths in this age group is significantly higher than the England average [2]. Rising suicide rates amongst men have been linked to economic recession and associated financial difficulties [3, 4]. A systematic review on personal unsecured debt and health showed a significant association between debt and mental ill-health, depression, substance misuse and suicide attempt or completion [5]. Whilst suicide is often medicalized in research literature, social, cultural, and economic factors are often involved, underscoring the importance of taking account of multiple factors in suicide prevention strategies [6]. Suicide prevention and support interventions often focus on psychological talking therapies [7, 8] which have been demonstrated as effective in reducing suicidal ideation [9]. It has also been highlighted that practical advice for debt and benefit issues might ease the mental health impact of any associated financial difficulties with debt, benefits and employment [10, 11]. Studies of interventions designed to mitigate effects of unemployment, debt or austerity showed mixed results [12–15]. Few of these studies targeted men [4], and tended to examine particular intervention types (e.g. employment support, debt counselling, welfare advice) rather than a holistic approach addressing individual needs and encompassing both financial and emotional support.

In the UK, the Department of Health and Social Care directed funding toward areas with high suicide rates to support suicide prevention and reduction schemes [16]. This supported the commissioning of Second Step, a local charity, to implement and run the ‘help for people with money, employment or housing problems’ project (now known as Hope) in the Bristol, North Somerset and South Gloucestershire area. Aimed at men aged 30 to 64, Hope provides face-to-face motivational interviewing sessions addressing acute distress felt by men who are suicidal [17] whilst also tackling debt, financial, employment or welfare difficulties. Phone calls and text messages supplement these sessions. Referrers include hospital mental health crisis teams, other statutory and voluntary agencies and self-referrals. Prior to implementation, a pilot randomised trial [18] determined the Hope service to be feasible and acceptable. The Hope service comprises of:

- Hope project workers, who provide psychologically informed support that encompasses a listening, non-judgemental, empowering and solution-focussed therapy approach. Project workers are trained in safety planning, suicide assessment and interventions, motivational interviewing and mental health e.g. dual diagnosis, personality disorders.
- Hope advice workers, who have specific training and qualifications as an adviser, in areas such as money, housing, employment, relationships, benefits and debt issues. Some workers were specialist debt case workers who are debt intermediaries, authorised and regulated by the insolvency service to apply for debt relief orders, following rules set out by the Financial Conduct Authority in the UK.

Further details of how the Hope service was structured, staff roles and training will be available in a separate qualitative article [Under review].

This article reports on how the Hope service has impacted on depression, suicidal ideation, financial self-efficacy of service users and their ability to manage financial, housing, employment and other difficulties, using quantitative and qualitative methods.

## Methods

### Quantitative evaluation

The quantitative evaluation used a prospective cohort study design. Second Step was commissioned to provide the Hope service to men aged 30–64 between November 2018 and October 2020. This evaluation covers the period between October 2018 and July 2020.

### Data collection

The evaluation used the Hope Project questionnaire (Additional file 1), comprised of PHQ-9 (Patient Health Questionnaire-9), [19] and FSES (Financial Self-Efficacy Scale) [20] questionnaires and questions about debt, employment, welfare benefits, and self-harm. The PHQ-9 measures depression severity, with a higher score indicating more severe depression. It has been shown to be a predictor of suicide attempts and deaths by suicide [21]. The FSES measures financial self-efficacy and reflects perceived ability to manage financial circumstances; a higher score indicates increased financial self-efficacy. The baseline questionnaires were administered by Hope workers during the first session to all service users, unless they declined to participate or were deemed to be in a highly vulnerable state or at imminent risk of suicide. Follow-up questionnaires were administered after six months, to all service users who completed the baseline questionnaire and attended their final session, unless they declined to complete the questionnaire or the Hope worker considered it inappropriate.

### Exposures and outcomes

The service includes face-to-face sessions with a Hope Project worker (number of sessions based on need) complemented by text messages and phone calls. One-to-one sessions were delivered primarily by telephone during the COVID-19 lockdown restrictions, although socially distanced face-to-face support sessions were provided wherever possible.

Hope service users were classified as having had low-intensity and moderate to high-intensity service use based on the number of contacts they had with the service (face-to-face, phone, and texts). Binary variables were generated categorising the number of each type of contact as low (1–3 contacts) or moderate/high (more than 3 contacts). Overall intensity of service use was categorised as low, or as moderate to high if service users had a moderate/high number of face-to-face contacts and a moderate/high number of phone or text contacts, or a moderate/high number of phone and text contacts (see Table 1).

**Table 1:** Overall intensity of service use based on number of face-to-face, phone and text contacts

Overall intensity of service use	Face-to-face sessions^a^	Phone calls^a^	Text Messages^a^
High intensity	moderate/high	moderate/high	moderate/high
High intensity	moderate/high	low	moderate/high
High intensity	moderate/high	moderate/high	low
Moderate intensity	moderate/high	low	low
Moderate intensity	low	moderate/high	moderate/high
Low intensity	low	low	moderate/high
Low intensity	low	moderate/high	low
Low intensity	low	low	low

^a^Low = 1–3 contacts, moderate/high = more than 3 contacts

The primary outcome was depression score, a continuous variable indicating the severity of depression as assessed by the PHQ-9 questionnaire. Secondary outcomes were suicidal ideation and financial self-efficacy. Suicidal ideation was measured as part of the PHQ-9 on a four-point scale and dichotomised as either yes (any suicidal ideation in the last 2 weeks) or no (no suicidal ideation in the last 2 weeks).

### Statistical analysis

Continuous variables were summarised using means and standard deviations (SD) and categorical data were summarised as numbers and percentages.

Generalised linear models were used to model the association between overall intensity of service use and the outcomes of interest. PHQ-9 and FSES scores at six months were regressed on the dichotomous explanatory variable of low-intensity service use or moderate/high-intensity service use. Logistic regression was conducted for suicidal ideation, regressed on low versus moderate/high-intensity service use.

Models were adjusted for baseline outcome measures (PHQ-9 score, suicidal ideation or FSES) and covariates: age, ethnicity, living conditions (living alone or with at least one partner or child), accommodation (renting, owned with mortgage, owned outright), number of benefits received, employment status (employed/unemployed), number of financial hardships (number of outgoings that are behind on payments), previous suicide attempt (yes/no), contact with mental health community services (yes/no), contact with financial/debt advise services (yes/no) and whether any period of follow-up fell after the national lockdown due to COVID-19 (yes/no). Models for PHQ-9 score and suicidal ideation were also adjusted for baseline FSES and models for FSES were adjusted for baseline PHQ-9 score.

Missing data for age, the missing components of the FSES, and lockdown (for service users missing follow-up date) were imputed using multiple imputation by chained equations, creating 20 imputed datasets. The imputation model included partially observed variables (age, lockdown, and FSES components), outcome variables, and all covariates included in each of the final models. Regression coefficients were combined across imputed datasets using Rubin’s rules. Regression analyses were repeated restricting to service users who had complete data on all covariates and the results were similar (data not shown).

All statistical analyses were performed in Stata version 16.1.

### Qualitative evaluation design and analysis

The qualitative study was underpinned by an interpretive approach which focuses on how people understand the world and how that informs their actions and experiences [22]. Semi-structured interviews were conducted with Hope service users. Recruitment took place from Aug-Sep 2020. Service users were identified by Second Step and supplied with a participant information sheet. If interested in participating they were invited to an interview at a prearranged time with JB or MF. Participants were purposefully sampled by age, ethnicity, mode of referral, level of debt, range of project workers they interacted with, and engagement with the service. One person declined involvement when introduced to the study, and another cancelled their interview due to illness. It was harder to contact those who had low or non-engagement with the service to invite them to be interviewed. Service users were offered a payment of £20 to recompense them for their time to participate. An interview distress protocol was developed with Second Step. When interviews were organised, the service user’s support worker would be informed and requested to keep time free after the interview in case the service user needed additional support [23]. Interviewers also had support workers’ contacts in case they had a concern about a service user’s welfare after interview. Where needed, interviewers (MF and JB) also contacted each other after interviews to reflect on the interview and discuss any issues arising. Service user interviews were part of a broader interview set, including Hope project and advice workers and NHS referrers (reported in [Under review]). The sampling strategy used information power to guide pragmatic decisions about numbers of interviewees, whereby data was analysed following study aims, specificity, dialogue and analysis [24].

A verbally recorded informed consent process preceded interviews which were conducted by telephone using a topic guide (Additional file 2) based on our research questions and associated literature. After interviews were completed, interviewers reminded service users that their support worker was available to talk if they wanted to connect with them. Data were reviewed throughout the data collection period at research team meetings until no new themes were emerging [25].

Interviews were audio recorded, transcribed, anonymised and checked for accuracy. We used a framework method of analysis [26], thematically analysing patterns and themes across the dataset. JB and MF each coded a sample of transcripts and jointly developed an initial coding framework. Following a further round of double coding (12% of transcripts), the framework was agreed and applied across the full dataset. Members of the study team met regularly to discuss analysis. The research adhered to the Consolidated criteria for Reporting Qualitative research guidelines for qualitative research.

### Mixed methods

We report the qualitative data related to and illustrative of the quantitative data which addresses the research aim of this paper. A separate qualitative paper addresses how Hope delivers its service in more detail [under review].

### Public involvement

Recruitment materials and interview questions were reviewed and edited by two patient and public involvement (PPI) members of the study team. Both attended project management meetings where data analysis and write up were discussed. Both were invited to be co-authors of this paper, and where accepted, reviewed the paper and provided comments.

## Results

### Quantitative study

#### Participants

A total of 413 people used the Hope service during the study period; 105 completed questionnaires at baseline (Additional file 3). Of these, 80 service users (76%) also completed questionnaires at six months follow-up. Table 2 shows baseline characteristics and engagement with the service of service users who completed both baseline and follow-up questionnaires, by intensity of service use. Service users, on average, had 5 face-to-face sessions (SD 2.7), 12 phone calls (SD 10.5), and 3 text messages (SD 3.9) with a Hope caseworker (Table 2). The mean age of service users was 47 years (SD 8.5). Eighty-three percent were of white ethnicity, 80% were unemployed, 80% were living alone, and 68% were living in rented accommodation. Around 25% received 3 or more benefits and 23% were behind on payments for 3 or more outgoings. Thirty-nine percent of service users had contact with community mental health services and 26% had contact with financial advice services. One service user was missing data for age, 6 were missing components of the FSES, and 2 were missing follow-up date.

**Table 2:** Baseline characteristics and engagement of service users, by intensity of service use

Variable	Low intensity (*n* = 16)		Moderate/high intensity (*n* = 64)		All (*n* = 80)	
	n/mean (SD)	%/range	n/mean (SD)	%/range	n/mean (SD)	%/range
Age^a^	46.3 (10.8)	30.9–58.3	47.6 (7.9)	31.0–64.0	47.3 (8.5)	30.9–64.0
Ethnicity^a^						
White	13	81.3	53	82.8	66	82.5
BAME	3	18.8	10	15.6	13	16.3
Not in employment^a^	13	81.3	51	79.7	64	80.0
Depression severity	21.0 (4.6)	9.0–27.0	20.0 (5.7)	5.0–27.0	20.2 (5.5)	5.0–27.0
Depression category:						
mild (5–9)	1	6.3	3	4.7	4	5.0
moderate (10–14)	0	0.0	11	17.2	11	13.8
moderately severe (15–19)	4	25.0	10	15.6	14	17.5
severe (20–27)	11	68.8	40	62.5	51	63.8
Financial self-efficacy^a^	11.0 (4.9)	6.0–20.0	11.1 (4.6)	6.0–24.0	11.1 (4.6)	6.0–24.0
Reported suicidal ideation	16	100.0	61	95.3	77	96.3
Had attempted suicide	3	18.8	34	53.1	37	46.3
No. benefits claimed:						
0	7	43.8	17	26.6	24	30.0
1–2	5	31.3	31	48.4	36	45.0
3–4	4	25.0	15	23.4	19	23.8
5+	0	0.0	1	1.6	1	1.3
Financial hardships^b^						
0	11	68.8	25	39.1	36	45.0
1–2	2	12.5	24	37.5	26	32.5
3–4	3	18.8	10	15.6	13	16.3
5+	0	0.0	5	7.8	5	6.3
Living alone	13	81.3	51	79.7	64	80.0
Accommodation^a^						
Owned	1	6.3	6	9.4	7	8.8
Mortgage/loan	1	6.3	8	12.5	9	11.3
Rented	11	68.8	43	67.2	54	67.5
Other	3	18.8	5	7.8	8	10.0
Had contact with community mental health services	4	25.0	27	42.2	31	38.8
Had contact with financial advice services	4	25.0	17	26.6	21	26.3
Had period of follow-up after lockdown^a^	5	31.3	14	21.9	19	23.8
Number of contacts with Hope service						
Face-to-Face	1.7 (1.1)	0–3	6.1 (2.2)	1–12	5.3 (2.7)	0–12
Phone calls	6.3 (6.4)	0–24	12.8 (11.0)	0–36	11.5 (10.5)	0–36
Text messages	0.9 (1.7)	0–6	3.9 (4.1)	0–15	3.3 (3.9)	0–15

^a^Age missing: 1 service user, ethnicity not known: 1, employment not known: 1, FSES score missing: 6, accommodation not known: 2, lockdown missing: 2

^b^no. of payments behind on

56.3% of low-intensity service users received one or more welfare benefits, compared to 73.4% of moderate/high-intensity service users (Table 2). Moderate to high-intensity service users were more likely to have had contact with community mental health services (42.1% vs 25.0%), be behind on payment for outgoings (60.9% vs 31.3%), and more likely to have had a previous suicide attempt (53.1% vs 18.8%). Baseline depression, suicide ideation, and financial self-efficacy was similar between low and moderate/high-intensity service users.

Of the 25 men who did not complete the follow-up questionnaire, 21(84%) were classified as high-intensity service users and the rest had at least one face-to-face session and between one and six additional contacts with the service via telephone or text message (see Additional file 3 for baseline characteristics).

#### Depression, suicidal ideation and financial self-efficacy

The mean depression scores for all service users decreased by 49% (10 points) from baseline to 6-month follow-up (Table 3). The number of service users reporting suicidal ideation on the PHQ-9 decreased by 55% (change in 42 service users). There was a similar reduction in mean depression scores and suicidal ideation amongst low-intensity and moderate to high-intensity service users.

**Table 3:** Difference in depression scores, suicidal ideation and FSES scores at 6-month follow-up compared to baseline

Outcome	Low (*n* = 16)		Moderate/high (*n* = 64)		Overall (*n* = 80)	
	mean/% difference (95% CI)	% change	mean/% difference (95% CI)	% change	mean/% difference (95% CI)	% change
PHQ-9 score	−11.3 (−14.3 to −8.4)	54%	−9.6 (−11.6 to −7.6)	48%	−10.0 (−11.7 to −8.3)	49%
Suicide ideation	−56.3% (−80.6% to −31.9%)	56%	−51.6% (−64.8% to −38.4%)	54%	−52.5% (−64.1% to −40.9%)	55%
FSES score^a^	4.1 (1.7 to 6.6)	35%	2.6 (1.3 to 3.8)	24%	2.9 (1.8 to 3.9)	26%

^a^FSES score was missing for 6 service users (3 low-intensity service users and 3 moderate/high-intensity service users)

Mean financial self-efficacy score at 6-month follow-up increased by 26% (2.9 points) compared to baseline. Greater increase in financial self-efficacy score was seen amongst low-intensity service users (35%) compared to moderate to high-intensity service users (24%).

#### Comparison between low and moderate to high-intensity service users

There was a 1.6 point greater reduction in mean depression score for moderate to high-intensity service use compared to low-intensity service use, after controlling for baseline score and confounding variables (Table 4), but the confidence interval (CI) was wide (95% CI = − 5.1 to 2.0). The moderate to high-intensity service use group had 60% lower odds of reporting suicidal ideation, but the estimate was imprecise (adjusted odds ratio 0.4; 95% CI = 0.1 to 2.3). There was little evidence of an association between intensity of service use and FSES score at 6 months (adjusted mean difference − 0.8; 95% CI = − 3.4 to 1.7; *p* = 0.52).

**Table 4:** Difference in outcomes at 6-month follow-up, between moderate to high and low-intensity service users

Variable	Effect estimate	95% CI	*p*-value
PHQ-9 score			
Adjusted mean difference	−1.6	−5.1 to 2.0	0.38
Unadjusted mean difference	0.7	−3.0 to 4.3	0.72
Suicidal ideation			
Adjusted odds ratio	0.4	0.1 to 2.3	0.31
Unadjusted odds ratio	1.0	0.3 to 3.0	1.00
Financial self-efficacy score			
Adjusted mean difference	−0.8	−3.4 to 1.7	0.52
Unadjusted mean difference	−1.2	−3.9 to 1.5	0.37

### Qualitative study

#### Participants

We interviewed 16 service users. Interviews lasted between 14 to 49 minutes (mean = 29 minutes). Where Hope Project and advice workers are referred to in data extracts, the gender terms her/she are used to maintain anonymity. Service user interviewee quotes are labelled from 09 to 24. The following sampling criteria were not met: people of non-white British ethnicity (*n* = 1) and those who did not engage with the service (*n* = 0). Several attempts were made to improve recruitment from these groups, but it was not possible in the project timeline. Table 5 outlines interview participant characteristics.

**Table 5:** Characteristics of service user interview participants

	Age range	Ethnic background	How referred^a^	Hope start date (by yearly quarters)
Each row relates a separate service user interviewed. No reference numbers given to maintain anonymity	61–70	White British	Mental health crisis team	Q1, 2020
	41–50	White British	Primary care	Q1, 2020
	51–60	White British	Job Centre	Not known
	51–60	White British	Job Centre	Q3,2019
	31–40	White British	Primary care	Q1, 2020
	41–50	White British	Local Charity	Q1, 2020
	51–60	Black British	Local Charity	Not Known
	51–60	White British	Mental health secondary services	Q3, 2019
	41–50	White British	Homeless outreach service	Q3, 2020
	51–60	White British	Primary care	Q1, 2020
	61–70	White British	Mental health secondary services	Q3, 2020
	31–40	White British	Mental health secondary services	Q1, 2020
	41–50	White British	Primary care	Q1, 2020
	51–60	White British	Mental health secondary services	Q1, 2019
	31–40	White British	Primary care	Q2, 2019
	31–40	White British	Mental health Crisis team	Not known

^a^Referral data was collected from interviewees during the interviews

#### Multiple factors that men faced

The qualitative data illustrates how men came to Hope with a range of issues including suicidal attempts, feelings, and thoughts; loss of employment; homelessness and housing problems; debt; problems with welfare benefits; addiction; bereavement; legal issues; custody battles; relationship breakdown; loneliness and isolation; and criminal injunctions. Most service users faced multiple issues, each overlapping and compounding the others. The multiplicity of issues contributed to their suicidal feelings:

“My nan died and for me it was like that was the last link to my family. I went downhill really quickly after that and, basically, yeah, she died, then the relationship I was in fell apart, which meant I lost my home from that and then with that and the other stresses, I lost the job that I had… It came to a head one night… I kind of hit rock bottom, basically took any tablets that I could find and then went down to the beach and just wanted to walk into the sea and keep walking.”(23)

“I was in a black hole, I was homeless, I was living on the streets … I was doing drugs … Yeah, just a lot happened then, like. I lost my girlfriend, I literally lost everything all in the space of, like, a week.”(24)

#### Service users experience of Hope

Priorities at the assessment session were to understand the most-pressing problems that were of concern and what emotional and practical support was needed, including referral to specialist Hope advice workers and other agencies. Almost all service users described how Hope workers were non-judgemental, supportive and easy to talk to, which enabled trust to develop:

“I picked up a trust very quick with ((name)), she was just so easy to get on with. I thought I might have had trust issues at first but after meeting her, I can't fault her, she was brilliant.”(16)

Participants spoke about feeling comfortable and being in control through their communications:

“(Project worker) just allowed me to flow and just go through what I needed to go through, which was, for my situation, the best thing I could have had. Cause the last thing I wanted was somebody to try to guide me and say, ‘You must do this or you should do this, have you considered this?’… I needed to talk and for somebody to listen and then just prod, suggest where I should go next.”(17)

The informal, non-judgemental approach of project workers enabled men to benefit and improve their situations through the service:

“What happened was, the more we spoke the more I could get things off my chest and the more she could understand where I was coming from and you know she’s just a fantastic listener and non-judgemental and all that organisation tries to do is you know to help you and also say ‘look there is a way out of the situation’.”(12)

Participants did not express negative experiences of using the Hope service, despite being asked about possible suggestions and improvements.

#### The scope of support

As we note above service users presented with multiple issues that ranged from mental health and suicidal attempts or ideation through to social and economic issues. Participants explained how Hope engaged with this multiplicity as a whole and how project workers helped make problems manageable, breaking down seemingly insurmountable difficulties into a series of achievable steps:

“When I first met up with (Hope Project worker) I was determined that life was at the end and I was fed-up and she turned it around. Like I couldn’t get a bank account and she got me a bank account, came into the bank and helped me out, and if she weren’t there I wouldn’t have got it because I didn’t have the relevant ID. I blow everything up into a really big problem and she cut it down into digestible chunks, if you like to call it that, and helped me through all of it. It’s been great.”(21)

Hope could advocate and directly engage with other institutions and organisations on the service user’s behalf. This could be making contact with benefits and housing organisations on the behalf of, or with clients, and accompanying them in meetings and assessments.

“She came to me with my appointments. She came to (health clinic) when I had to see the psychiatrist…. She took me we went down to the council and discussed some sort of programme to get me out of the hostel I was in.” (Also went to personal independence payment (PIP) assessment)(12)

Specifically, funded Hope advice workers gave quick access to financial support and advice and tackled debt issues which helped to relieve anxiety and stress:

“I’m now in the process of going into a debt recovery order and what have you, so they’ve helped me financially and with my depression and mental health.”(21)

“There was still 100’s and 100’s of pounds that they were expecting me to pay… it was giving me a lot of anxiety and (advice worker’s name), at the end of the day, she got it squashed, they said ‘you don’t have to pay anything’, which was fantastic.”(20)

The ability to engage holistically with a range of services and agencies made Hope different in the eyes of service users.

“It’s not just when she was coming round it’s not just the emotional and the issue I was dealing with in my health it was more generalised as well which I think was a great thing because other services I’ve used haven’t had that overall approach and I think mental health needs to be dealt with overall”(19).

“They sorted my life out, I don’t know what else I could say, my life at the time, I had my money slashed to bits which I don’t know how I survived plus I felt myself sinking into depression. Between them, in completely different ways, (Project worker) and (Advice worker) have put me back on track. I don’t know where I was going, I really don’t, I wasn’t sleeping, I wasn’t eating, I was a bit of a mess. They certainly helped me.”(16).

Several used the GP as a point of comparison highlighting how Hope gave them the time and opportunity to describe the issues they faced and their overall mental health in a way that was not possible with a GP:

“When I went to the doctors I felt like I was just talking to, I don’t know how to describe it, a brick wall, like the doctors didn’t even look up to chat to you like they do, it’s like they needed to see the next patient”(21)

The ability to be open and honest in communications between service users and project workers was also linked to Hope being seen as independent with no other agenda than to help and support:

“I felt that they [Hope] wanted to help, whereas I felt like the other ones, if it went the wrong way, would lock me up.”(23)

#### Impacts of Hope: empowering change

The quantitative results highlighted reductions in depression and suicidal ideation scores and improvement in financial self-efficacy. These were evident in the qualitative data. Several service users stated how Hope saved their lives and helped when they were feeling suicidal:

“To cut a long story short if it weren’t for the Hope Project and ((name)) I would not have made it back out through the rabbit hole, I was done. And I can’t thank the organisation enough because at the end of the day I’ve had several friends who’ve committed suicide which is a terrible thing but what I know for one thing is sure if they were being given the same amount of help which I was given I’m sure that some of them would still be alive today that’s how important the Hope Project is. I mean to cut a long story short, the Hope Project saves lives (original emphasis) that’s a fact”(12).

“They basically kept me alive… I can hand on heart say I think they saved me… it’s brilliant.”(21)

Service users explained how Hope staff supported them at times of suicidal feelings:

“There was a couple of times that I did feel suicidal, because you had that person there and they understand and they just listen, yeah, it’s good to have people that have got experience… it certainly helps”.(18)

Service users also described how their experience with the Hope service gave them confidence to manage setbacks in the present and future:

“I’ve just lost my job again. I’ve just been basically dismissed because the company is folding because of the COVID and literally this is my last day today so I’m back in that situation of not having a job. It’s a lot easier than what it was before because before I can’t point out enough that I was in serious depression and thoughts of suicide was coming into my head because you just feel like you’ve failed if you know what I mean”.(18)

“Before I would have just buried my head in the sand until that had gone away or I would have ended up doing something drastic which I would have regretted doing. When the Hope service came in, they sat me down and at all the sessions by the time the session had finished I would be able to actually put situations into context and actually be able to manage them in a more positive light and deal with them in a more positive way.”(22)

As time progressed for people, some felt more empowered to represent themselves in dealing with external agencies:

“I definitely am in a much better place to represent myself now. Usually now, if I’ve got to do something, I’ll let (Project worker) know that I’m doing it, but I won’t ask her to help me with it.”(23)

## Discussion

### Summary of results

Mean depression score and number of service users with suicidal ideation at 6 months were reduced by at least 49% compared to baseline. There was no evidence of a difference in this reduction between low-intensity service users and moderate to high-intensity service users. There was a 26% increase in financial self-efficacy, with a larger increase amongst low-intensity service users (35%) compared to moderate to high-intensity service users (24%). However, due to imprecision in the estimates of the effect of increased intensity of service use, it was not possible to rule out null or negative effects.

The improvements across the quantitative parameters were supported by the qualitative data in which accounts of the life-situation by men using Hope were much improved following service engagement. Men described suicidal intentions and actions in depicting their circumstances upon presenting to Hope which were usually accompanied by a range of social and economic difficulties. By working with Hope, who gave practical and emotional support, they were able to move forward and begin to tackle their life challenges with more confidence. Key to this progress was the relationship between service users and project workers; the nature of the sessions between them facilitated establishment of trust which enabled men to feel comfortable in opening-up and discussing their problems, an experience they had not always had with other health services, such as GP services; and integrated support to tackle both emotional and practical difficulties.

### Comparison with other studies

Our results highlight that a key part of the Hope service is the combination of mental health and social support provided by project workers, alongside expert financial advice of funded specialist advice workers (detailed description of these roles will be available in a forthcoming qualitative paper currently under review). Previous studies have evaluated services which combine these aspects, to some degree, but results have been inconclusive and few have focused specifically on suicide as an outcome.

Interviews with vulnerable people who had self-harmed due to financial difficulty or who were struggling financially following the Great Recession (2008–9) showed that access to free financial advice could help mitigate the impact of financial difficulties on mental health [10]. One intervention offering free telephone advice from National Debtline to people in debt in England and Wales, provided immediate advice and assistance relating to emergency issues (e.g. threats of bailiff action) and help to resolve longer-term financial problems, though did not incorporate mental health support as provided by the Hope service. Findings from this trial showed little evidence of a reduction in anxiety or indebtedness [27]. Another study of the effect of co-locating welfare advice services with primary care showed evidence of improved mental health and reduced financial strain for women and black British recipients, but men, in general, did not show the same level of improvement [28]. Whilst co-located welfare advice in general practice may reach people who may not otherwise seek welfare advice, the intervention did not specifically combine such advice with mental health support in the way that the Hope service does. A review of psychosocial and policy interventions to mitigate the effects of poverty and inequality on mental health found strong evidence for the effectiveness and cost-effectiveness of mental health promotion activities targeting people at risk but a lack of conclusive evidence for service-based (e.g. health services, social prescribing, debt advice and financial counsellors) or community interventions [29]. Although some of these studies have shown positive results, interventions tend to focus on either financial advice or mental health promotion rather than a combination and tend not to focus on men, or on suicide as an outcome. The positive findings from this evaluation of the Hope service suggests a beneficial impact of the more holistic approach to providing combined financial and mental health support.

A systematic review which examined rates of contact with primary and mental health care prior to suicide found that contact with primary health care was common in the final month before death, highlighting the importance of suicide prevention strategies linked to primary care [30]. Yet research has illustrated how GPs, who remain the first point of contact for people with mental health problems, may have insufficient time, tools and resources to appropriately support people with complex psycho-social needs [31]. Educating primary care professionals on suicide prevention is beneficial [32], but improved connections between community mental health services and primary care are also key [33, 34]. This is supported by a systematic review of the role of GPs in the management of patients who self-harm, identifying a need for GP training, enhanced communication between primary care and mental health teams and enhanced service provision [35]. Shortages in time, funding and resources such as patient-liaison and community services or in-practice self-harm services were identified as barriers to GP management [35]. Almost a third of our interviewees were referred directly via primary care (Table 5), which enabled a community based, enhanced service provision, that was not available elsewhere.

The role of the project worker and their ability to create a non-judgemental and trusting relationship was also critical. Previous studies have highlighted the importance of the development of trust and respect in the relationship between professionals and service users, of not feeling judged, and the ability to communicate empathy [4, 34, 36, 37]. These factors featured strongly in the relationship between Hope Project workers and service users, augmented by direct engagement with advice-giving and/or signposting to appropriate agencies whilst retaining an ongoing relationship with the project worker. The qualitative findings illustrate how an informal, non-judgemental and supportive approach enabled men to open up and share concerns and suicidal feelings. This reaffirms research that highlights the importance and value of informal, community-based support [4, 38, 39] which are not characterised by the same level of unequal social relationships that may occur when talking with medical professionals and psychiatrists [4, 38, 39].

### Strengths

A major strength of the quantitative evaluation is the prospective collection of data and measurement of outcomes. The data were also collected using standardised and validated questionnaires.

The mixed methods approach also strengthened the findings by (a) giving depth and detail to the quantitative data around the service user experience and journey through the service, and (b) each data set told a similar story thereby increasing confidence in the findings.

### Limitations

Though the reported crude differences, between baseline and follow-up values appear impressive, this was not a randomised study and we could not account for missing or unknown confounders. Modelling of high-intensity service users compared to low-intensity service users found no evidence of a reduction in depression or suicide ideation associated with intensity of service use. All individuals included in our models have engaged with the Hope service to some extent and we could not compare with individuals who have not used the service. Nevertheless, we would expect a higher effect if the service users were compared to a control group who had no engagement with the service.

Although 413 men used the service during the study period, only 105 completed the baseline questionnaire. It is possible therefore that our sample does not include service users who might be at greater risk, which might limit the generalisability of our findings to service users with less severe illness. These users are, however, less likely pass the threshold for specialist secondary care services and more likely to continue accessing these community-based services. The small sample size also meant that the analysis was underpowered to investigate a dose-response relationship. Despite the use of multiple imputation to increase precision in the estimates, it was not possible to draw strong conclusions about the effectiveness of increased intensity of service use and the estimates could not rule out null or negative effects.

There was some attrition from the study, with 25 men who did not complete the follow-up questionnaire. If these men experienced an increase in depression scores or suicidal ideation during the study period, this may bias our results towards an overestimation of the true effectiveness of the service. However, the precise reasons for not completing the follow-up questionnaire are unknown and may vary. The sample for the qualitative component did not meet some of our targets concerning diversity. Service users from Black, Asian and other ethnic communities were under-represented. Service users who had low/non-engagement with the service were more difficult to contact which may have resulted in negative views or experiences of Hope not being represented in the findings.

## Conclusion

Our findings suggest that engagement with the Hope service helped to reduce men’s depression and suicidal ideation and improved financial self-efficacy through the unique combination of one-to-one practical and emotional support offered. Whilst general advice services may provide practical, financial, and legal support, and mental health services provide emotional support, Hope enables access to both, addressing an identified gap in service [40]. Service users engaged with Hope in a way that they had not been able to with other support services. This indicates that Hope is serving a previously underserved population and playing a key role in the overall field of mental health care provision. It complements existing services in the NHS and in the community by targeting a particularly high-risk group of men and helps prevent the escalation of their situation.

## Supplementary Information

**Additional file 1.**

**Additional file 2.**

**Additional file 3.**

## Data Availability

The quantitative data for the study were collected by the service provider, Second Step, who retains ownership of the data. The study was underpinned by a data sharing agreement between Second Step and the University of Bristol (as joint controllers), and in accordance with the study subjects’ consent. Under this agreement, no data can be shared with any third party, but access to the data can be requested from Second Step. The qualitative data collected and analysed during the study cannot be made publicly available for confidentiality reasons, but they can be discussed with the corresponding author on reasonable request.

## Abbreviations

FSES: Financial self-efficacy scale
PHQ-9: Patient Health Questionnaire-9
PPI: Patient and public involvement
CI: Confidence interval
PIP: Personal independence payment

## References

[1] Suicides in the UK: 2018 registrations [Online]. [https://www.ons.gov.uk/peoplepopulationandcommunity/birthsdeathsandmarriages/deaths/bulletins/suicidesintheunitedkingdom/2018registrations]. Accessed 23 Mar 2022.

[2] Bristol JSNA Health and wellbeing profile 2021/22 [https://www.bristol.gov.uk/policies-plans-strategies/jsna-data-profile]. Accessed 23 Mar 2022.

[3] Ibrahim S, Hunt IM, Rahman MS, Shaw J, Appleby L, Kapur N (2019). Recession, recovery and suicide in mental health patients in England: time trend analysis. Br J Psychiatry.

[4] Struszczyk S, Galdas PM, Tiffin PA (2019). Men and suicide prevention: a scoping review. J Ment Health.

[5] Richardson T, Elliott P, Roberts R (2013). The relationship between personal unsecured debt and mental and physical health: a systematic review and meta-analysis. Clin Psychol Rev.

[6] Nugent AC, Ballard ED, Park LT, Zarate CA (2019). Research on the pathophysiology, treatment, and prevention of suicide: practical and ethical issues. BMC Psychiatry.

[7] Gooding PA, Pratt D, Awenat Y, Drake R, Elliott R, Emsley R, Huggett C, Jones S, Kapur N, Lobban F (2020). A psychological intervention for suicide applied to non-affective psychosis: the CARMS (cognitive AppRoaches to coMbatting suicidality) randomised controlled trial protocol. BMC Psychiatry.

[8] McCabe R, Garside R, Backhouse A, Xanthopoulou P (2018). Effectiveness of brief psychological interventions for suicidal presentations: a systematic review. BMC Psychiatry.

[9] Schneider RA, Chen SY, Lungu A, Grasso JR (2020). Treating suicidal ideation in the context of depression. BMC Psychiatry.

[10] Barnes MC, Donovan JL, Wilson C, Chatwin J, Davies R, Potokar J, Kapur N, Hawton K, O’Connor R, Gunnell D (2017). Seeking help in times of economic hardship: access, experiences of services and unmet need. BMC Psychiatry.

[11] Barnes MC, Gunnell D, Davies R, Hawton K, Kapur N, Potokar J, Donovan JL (2016). Understanding vulnerability to self-harm in times of economic hardship and austerity: a qualitative study. BMJ Open.

[12] Allmark P, Baxter S, Goyder E, Guillaume L, Crofton-Martin G (2013). Assessing the health benefits of advice services: using research evidence and logic model methods to explore complex pathways. Health Soc Care Community.

[13] Audhoe SS, Hoving JL, Sluiter JK, Frings-Dresen MH (2010). Vocational interventions for unemployed: effects on work participation and mental distress. A systematic review. J Occup Rehabil.

[14] Dobbie L, Gillespie M (2010). The health benefits of financial inclusion: a literature review. Report for NHS Greater Glasgow and Clyde.

[15] Moore TH, Kapur N, Hawton K, Richards A, Metcalfe C, Gunnell D (2017). Interventions to reduce the impact of unemployment and economic hardship on mental health in the general population: a systematic review. Psychol Med.

[16] Mental Health Taskforce: The Five Year Forward View for Mental Health. 2016. https://www.england.nhs.uk/mental-health/taskforce/.

[17] Miller WR, Moyers TB (2017). Motivational interviewing and the clinical science of Carl Rogers. J Consult Clin Psychol.

[18] Barnes MC, Haase AM, Scott LJ, Linton MJ, Bard AM, Donovan JL, Davies R, Dursley S, Williams S, Elliott D (2018). The help for people with money, employment or housing problems (HOPE) intervention: pilot randomised trial with mixed methods feasibility research. Pilot Feasibility Stud.

[19] Kroenke K, Spitzer RL, Williams JB (2001). The PHQ-9: validity of a brief depression severity measure. J Gen Intern Med.

[20] Lown JM (2011). 2011 outstanding AFCPE® conference paper: development and validation of a financial self-efficacy scale. J Financ Couns Plan.

[21] Rossom RC, Coleman KJ, Ahmedani BK, Beck A, Johnson E, Oliver M, Simon GE (2017). Suicidal ideation reported on the PHQ9 and risk of suicidal behavior across age groups. J Affect Disord.

[22] Schwandt T (2000). Three epistemological stances for qualitative inquiry: interpretivism, hermeneutics, and social constructionism. In: handbook of qualitative research volume 2, edn. Edited by Denzin N, Lincoln Y.

[23] Littlewood DL, Harris K, Gooding P, Pratt D, Haddock G, Peters S: Using my demons to make good: the short- and long-term impact of participating in suicide-related research. Arch Suicide Res 2021, 25(2):315–339. 10.1080/13811118.2019.1663330 31544686

[24] Malterud K, Siersma VD, Guassora AD (2016). Sample Size in Qualitative Interview Studies:Guided by Information Power. Qual Health Res.

[25] Sandelowski M (1995). Sample size in qualitative research. Res Nurs Health.

[26] Gale NK, Heath G, Cameron E, Rashid S, Redwood S (2013). Using the framework method for the analysis of qualitative data in multi-disciplinary health research. BMC Med Res Methodol.

[27] Pleasence P, Balmer NJ. Changing Fortunes: Results from a Randomized Trial of the Offer of Debt Advice in England and Wales. J Empir Legal Stud. 2007;4(3):651–73.

[28] Woodhead C, Khondoker M, Lomas R, Raine R (2017). Impact of co-located welfare advice in healthcare settings: prospective quasi-experimental controlled study. Br J Psychiatry.

[29] Wahlbeck K, Cresswell-Smith J, Haaramo P, Parkkonen J (2017). Interventions to mitigate the effects of poverty and inequality on mental health. Soc Psychiatry Psychiatr Epidemiol.

[30] Stene-Larsen K, Reneflot A (2019). Contact with primary and mental health care prior to suicide: a systematic review of the literature from 2000 to 2017. Scand J Public Health.

[31] Leavey G, Mallon S, Rondon-Sulbaran J, Galway K, Rosato M, Hughes L (2017). The failure of suicide prevention in primary care: family and GP perspectives – a qualitative study. BMC Psychiatry.

[32] Mann JJ, Michel CA, Auerbach RP (2021). Improving suicide prevention through evidence-based strategies: a systematic review. Am J Psychiatr.

[33] Elzinga E (2020). de Kruif AJTCM, de Beurs DP, Beekman ATF, Franx G, Gilissen R: engaging primary care professionals in suicide prevention: a qualitative study. PLoS One.

[34] O’Donnell P, Tierney E, O’Carroll A, Nurse D, MacFarlane A (2016). Exploring levers and barriers to accessing primary care for marginalised groups and identifying their priorities for primary care provision: a participatory learning and action research study. Int J Equity Health.

[35] Mughal F, Troya MI, Dikomitis L, Chew-Graham CA, Corp N, Babatunde OO (2020). Role of the GP in the management of patients with self-harm behaviour: a systematic review. Br J Gen Pract.

[36] Nicholas A, Pirkis J, Reavley N. What responses do people at risk of suicide find most helpful and unhelpful from professionals and non-professionals? J Ment Health. 2020:1–10. Published online September 15, 2020. 10.1080/09638237.2020.1818701 32930018

[37] Parker D, Byng R, Dickens C, McCabe R (2020). “Every structure we're taught goes out the window”: general practitioners' experiences of providing help for patients with emotional concerns. Health Soc Care Community.

[38] Hung P, Busch SH, Shih Y-W, McGregor AJ, Wang S (2020). Changes in community mental health services availability and suicide mortality in the US: a retrospective study. BMC Psychiatry.

[39] Chandler A. Masculinities and suicide: unsettling ‘talk’ as a response to suicide in men. Crit Public Health. 2021:1–10. Published online April 08, 2021.

[40] Gunasinghe C, Gazard B, Aschan L, MacCrimmon S, Hotopf M, Hatch SL (2018). Debt, common mental disorders and mental health service use. J Ment Health.

