# Supplementary material for: Preventing male suicide through a psychosocial intervention that provides psychological support and tackles financial difficulties: a mixed method evaluation
Source: BMC Psychiatry. 2022 May 13;22:333. doi: 10.1186/s12888-022-03973-5 (PMC9103598; doi:10.1186/s12888-022-03973-5)
Supplement: Supplementary file 1 — Additional file 1. [file 12888_2022_3973_MOESM1_ESM.docx]

## Hope baseline and follow-up questionnaires


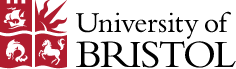


**CONFIDENTIAL**

**Baseline Measures: Intervention**

**Help for peOPle with money, Employment or benefit problems**

Thank you for taking part in our research. This questionnaire should take 10-15 minutes to complete. We are interested in finding out about you and your health, emotions and finances. We understand it’s not always easy to choose an option that describes exactly what you are feeling, if you are not sure, please choose the response that comes closest to how you feel. Please answer all questions. All your responses will be confidential and not released to anyone except the research team.

If you need help with any questions please contact Maria Barnes tel: 0117 3313929

Study ID ________________________________

Date of birth ________/________/19____________

Gender Male

Female

Researcher ________________________________

Date ­­­­­­­­­­­­­­­­­­­­­­­­­­­­­­­­­­­­­­­­­­­­­­____/_____/______

**Section A Your mood**

1. **These questions are about how you feel and how things have been with you during the past 2 weeks. For each question, please give one answer that comes closest to the way you have been feeling. (Please tick one box for each question).**

| **Over the last 2 weeks, how often have you been bothered by any of the following problems?** | **Not at all** | **Several Days** | **More than half the days** | **Nearly every day** |
| --- | --- | --- | --- | --- |
| a) Little interest or pleasure in doing things |  |  |  |  |
| b) Feeling down, depressed or hopeless |  |  |  |  |
| c) Trouble falling or staying asleep, or staying asleep too much |  |  |  |  |
| d) Feeling tired or having little energy |  |  |  |  |
| e) Poor appetite or overeating |  |  |  |  |
| f) Feeling bad about yourself, or that you are a failure, or have let yourself or your family down |  |  |  |  |
| g) Trouble concentrating on things, such as reading the newspaper or watching television |  |  |  |  |
| h) Moving or speaking so slowly that other people could have noticed. Or the opposite – being so fidgety or restless that you have been moving around a lot more than usual |  |  |  |  |
| i) Thoughts that you would be better off dead, or of hurting yourself in some way |  |  |  |  |

**Section B Your health and well-being**

1. **By placing a tick in one box in each group below, please indicate which statements best describe your own health state today**:

|  | **Mobility** | | |
| --- | --- | --- | --- |
|  | I have no problems in walking about | |  |
|  | I have slight problems in walking about | |  |
|  | I have some moderate problems in walking about | |  |
|  | I have severe problems in walking about | |  |
|  | I am unable to walk about | |  |
|  | **Self-Care** | | |
|  | I have no problems washing or dressing myself | |  |
|  | I have slight problems washing or dressing myself | |  |
|  | I have moderate problems washing or dressing myself | |  |
|  | I have severe problems washing or dressing myself | |  |
|  | I am unable to wash or dress myself | |  |
|  | **Usual Activities** **(e.g. work, study, housework, family or leisure activities)** | | |
|  | I have no problems doing my usual activities | |  |
|  | I have slight problems doing my usual activities | |  |
|  | I have moderate problems doing my usual activities | |  |
|  | I have severe problems doing my usual activities | |  |
|  | I am unable to do my usual activities | |  |
|  | **Pain / Discomfort** | | |
|  | I have no pain or discomfort | |  |
|  | I have slight pain or discomfort | |  |
|  | I have moderate pain or discomfort | |  |
|  | I have severe pain or discomfort | |  |
|  | I have extreme pain or discomfort | |  |
|  | **Anxiety / Depression** | | |
|  | I am not anxious or depressed | |  |
|  | I am slightly anxious or depressed | |  |
|  | I am moderately anxious or depressed | |  |
|  | I am severely anxious or depressed | |  |
|  | I am extremely anxious or depressed | |  |
|  | |  |  |
|  | |  |  |

**Section C Your feelings**

1. **The following questions ask you about feelings of anxiety. For each question please give the answer that comes closest to the way you have been feeling during the past 2 weeks. (Please tick one box for each question).**

| **Over the last 2 weeks, how often have you been bothered by the following problems?** | **Not at all** | **Several days** | **More than half the days** | **Nearly every day** |
| --- | --- | --- | --- | --- |
| a) Feeling nervous, anxious or on edge |  |  |  |  |
| b) Not being able to stop or control worrying |  |  |  |  |
| c) Worrying too much about different things |  |  |  |  |
| d) Trouble relaxing |  |  |  |  |
| e) Being so restless that it is hard to sit still |  |  |  |  |
| f) Becoming easily annoyed or irritable |  |  |  |  |
| g) Feeling afraid as if something awful might happen |  |  |  |  |

1. **Thinking back to when you were recently admitted to the BRI (Bristol Royal Infirmary), what were the main three factors that contributed to your crisis / self-harm?**
2. ………………………………………………………………………………………………………………………………………..
3. …………………………………………………………………………………………………………………………………………
4. ……………………………………………………………………………………………………………………………………………

**Section D The following questions ask about financial stresses and strains in your life, and the help you receive.**

| **AFFORDABILITY, Please tick one box for each question** | | | | |
| --- | --- | --- | --- | --- |
| 1. **How often does it happen that you do not have enough money to:** | Often | Sometimes | Never | Don’t know |
| Afford the kinds of food that you or your family should have? |  |  |  |  |
| Afford the kinds of clothing that you or your family should have? |  |  |  |  |
| 1. **How often does it happen that you find it difficult to:** | Often | Sometimes | Never | Don’t know |
| Pay bills for gas and/or electricity? |  |  |  |  |
| Pay bills for the telephone? |  |  |  |  |
| Pay your rent or mortgage? |  |  |  |  |

| 1. **How dissatisfied or satisfied are you with the income of your household?** | | | | | | |
| --- | --- | --- | --- | --- | --- | --- |
| Completely satisfied | Mostly satisfied | Somewhat satisfied | Neither satisfied nor dissatisfied | Somewhat dissatisfied | Mostly dissatisfied | Completely dissatisfied |
|  |  |  |  |  |  |  |

| 1. In the next 2 months how much do you anticipate that you or your family will experience financial hardships such as inadequate food, housing clothing or heating? | | | | |
| --- | --- | --- | --- | --- |
| Never | Rarely | Sometimes | Often | Always |
|  |  |  |  |  |

| 1. **Please respond to the following statements by ticking just one box:** | **Exactly true** | **Moderately true** | **Hardly true** | **Not at all true** |
| --- | --- | --- | --- | --- |
| It is hard to stick to my spending plan when unexpected expenses arise |  |  |  |  |
| It is challenging to make progress towards my financial goals |  |  |  |  |
| When unexpected expenses occur I usually have to use credit |  |  |  |  |
| When faced with a financial challenge, I have a hard time figuring out a solution |  |  |  |  |
| I lack confidence in my ability to manage my finances |  |  |  |  |
| I worry about running out of money in retirement |  |  |  |  |

**Section E Your Work, Social & Financial Situation**

| **EMPLOYMENT**   1. **Are you currently doing paid work of any sort? [tick one]** | **Yes** | **No** |
| --- | --- | --- |
| If **Yes** (you are currently working):   **How many hours do you work per week on average?** ……………. **hours   How long have you been working in your current job?** ………….... **years** ……………**months** | | |
| If **No** (you are not currently working):  **How long have you been out of work?** ………………. **years** ………… **months**  **What is the reason you are out of work?** | | |

1. **What is (was) the main activity of your employer or business?**

(For example PRIMARY EDUCATION, REPAIRING CARS, CONTRACT CATERING, COMPUTER SERVICING. Write GOVERNMENT - if you are/were civil servant, write LOCAL GOVERNMENT - if you are/were a local government officer)

……………………………………………………………………………………………………………………………………………….

1. **What is (was) the name of the organisation you work (worked) for?**

If you are (were) self-employed in your organisation, write in the business name. If you are (were) self-employed, freelance or work (worked) for a private individual, write No Organisation.

……………………………………………………………………………………………………………………………………………….

1. **What is (was) your specific job title?**

(For example PRIMARY SCHOOL TEACHER, DISTRICT NURSE, STRUCTURAL ENGINEER, CAR MECHANIC. Do not state your grade or payband)

……………………………………………………………………………………………………………………………………………….

1. **Briefly describe what you do (did) in your main job:**

……………………………………………………………………………………………………………………………………………….

1. **Do (did) you supervise any employees?** **Yes**  **No**

(Supervision involves overseeing work of other employees on a day-to-day basis)

| **BENEFITS**   1. **Do you or your partner receive any of the following?  Please tick ALL that apply** | **You** | **Your**  **Partner** |
| --- | --- | --- |
| Job seekers allowance |  |  |
| Employment support allowance |  |  |
| Disability living allowance |  |  |
| Attendance allowance |  |  |
| Carers allowance |  |  |
| Council tax benefit |  |  |
| Housing benefit |  |  |
| Working tax credit |  |  |
| Income support |  |  |
| Statutory sick pay |  |  |
| Child benefit |  |  |
| Don’t know |  |  |
| **Other (please specify)** | | |

| **DEBT AND OTHER HARDSHIPS**   1. **Are you or your partner behind on payments for any of the following? Please tick ALL that apply** | **You** | **Your Partner** |
| --- | --- | --- |
| Mortgage |  |  |
| Rent |  |  |
| Electricity/gas/water |  |  |
| Council tax |  |  |
| Benefits (e.g. overpayments of tax credits) |  |  |
| Credit card |  |  |
| Hire Purchase (e.g. loan for care or other possessions) |  |  |
| Student loans |  |  |
| Business loans |  |  |
| Loans from family or friends |  |  |
| Short term cash loan (e.g. payday or other lenders) |  |  |
| Taxes |  |  |
| **Other (please specify)** | | |

| 1. **Which of the following organisations have you ever used? Please tick ALL that apply** | |
| --- | --- |
| General advice centres (e.g. Citizens Advice Bureau or local advice centres) |  |
| Housing benefit / homelessness services (e.g. Shelter) | ☐ |
| Debt advice services (e.g. Talking Money and Pennywise) | ☐ |
| Alcohol / drug advice services (e.g. Bristol Roads) | ☐ |
| Mental health charities (e.g. MIND and Samaritans) | ☐ |
| Foodbanks | ☐ |
| Other advice or support for financial problems |  |
| **Any other (please specify)** | |

**Section F Home and Family**

| 1. **Who is in your household?** | | **How many** |
| --- | --- | --- |
| Partner |  |  |
| Children (under 16) |  |  |
| Children (16 or over) |  |  |
| Parents |  |  |
| **Other (please specify)** | | |

| 1. **Is the accommodation in which you live:  (tick as appropriate)** | |
| --- | --- |
| Owned outright by you or your partner |  |
| Owned with mortgage/loan by you or your partner |  |
| Rented |  |
| **Other (please specify)** | |

**Finally**

| 1. **Can you please select the ethnic group that you feel best describes your cultural background?** | |
| --- | --- |
| White |  |
| Mixed/multiple |  |
| Asian/Asian British |  |
| Black/African/Caribbean/Black British |  |
| Other |  |

**Contact details**

May we have a friend or family’s contact details in case we are unable to get hold of you?

Address

Telephone

Email

**MANY THANKS FOR ANSWERING THESE QUESTIONS**

**Appendix 1.3**


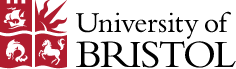


**CONFIDENTIAL**

**Follow-up Questionnaire**

**Help for peOPle with money, Employment or benefit problems**

Thank you for taking part in our research. This questionnaire should take 10-15 minutes to complete. We are interested in finding out about you and your health, emotions and finances. We understand it’s not always easy to choose an option that describes exactly what you are feeling, if you are not sure, please choose the response that comes closest to how you feel. Please answer all questions. All your responses will be confidential and not released to anyone except the research team.

If you need help with any questions please contact Maria Barnes tel: 0117 3313929

Study ID ________________________________

Date of birth ________/________/19____________

Gender Male

Female

Researcher ________________________________

Date ­­­­­­­­­­­­­­­­­­­­­­­­­­­­­­­­­­­­­­­­­­­­­­____/_____/______

**Section A Your mood**

1. **These questions are about how you feel and how things have been with you during the past 2 weeks. For each question, please give one answer that comes closest to the way you have been feeling. (Please tick one box for each question).**

| **Over the last 2 weeks, how often have you been bothered by any of the following problems?** | **Not at all** | **Several Days** | **More than half the days** | **Nearly every day** |
| --- | --- | --- | --- | --- |
| a) Little interest or pleasure in doing things |  |  |  |  |
| b) Feeling down, depressed or hopeless |  |  |  |  |
| c) Trouble falling or staying asleep, or staying asleep too much |  |  |  |  |
| d) Feeling tired or having little energy |  |  |  |  |
| e) Poor appetite or overeating |  |  |  |  |
| f) Feeling bad about yourself, or that you are a failure, or have let yourself or your family down |  |  |  |  |
| g) Trouble concentrating on things, such as reading the newspaper or watching television |  |  |  |  |
| h) Moving or speaking so slowly that other people could have noticed. Or the opposite – being so fidgety or restless that you have been moving around a lot more than usual |  |  |  |  |
| i) Thoughts that you would be better off dead, or of hurting yourself in some way |  |  |  |  |

**Section B Your health and well-being**

1. **By placing a tick in one box in each group below, please indicate which statements best describe your own health state today**:

|  | **Mobility** | | |
| --- | --- | --- | --- |
|  | I have no problems in walking about | |  |
|  | I have slight problems in walking about | |  |
|  | I have some moderate problems in walking about | |  |
|  | I have severe problems in walking about | |  |
|  | I am unable to walk about | |  |
|  | **Self-Care** | | |
|  | I have no problems washing or dressing myself | |  |
|  | I have slight problems washing or dressing myself | |  |
|  | I have moderate problems washing or dressing myself | |  |
|  | I have severe problems washing or dressing myself | |  |
|  | I am unable to wash or dress myself | |  |
|  | **Usual Activities** **(e.g. work, study, housework, family or leisure activities)** | | |
|  | I have no problems doing my usual activities | |  |
|  | I have slight problems doing my usual activities | |  |
|  | I have moderate problems doing my usual activities | |  |
|  | I have severe problems doing my usual activities | |  |
|  | I am unable to do my usual activities | |  |
|  | **Pain / Discomfort** | | |
|  | I have no pain or discomfort | |  |
|  | I have slight pain or discomfort | |  |
|  | I have moderate pain or discomfort | |  |
|  | I have severe pain or discomfort | |  |
|  | I have extreme pain or discomfort | |  |
|  | **Anxiety / Depression** | | |
|  | I am not anxious or depressed | |  |
|  | I am slightly anxious or depressed | |  |
|  | I am moderately anxious or depressed | |  |
|  | I am severely anxious or depressed | |  |
|  | I am extremely anxious or depressed | |  |
|  | |  |  |
|  | |  |  |

**Section C Your feelings**

1. **The following questions ask you about feelings of anxiety. For each question please give the answer that comes closest to the way you have been feeling during the past 2 weeks. (Please tick one box for each question).**

| **Over the last 2 weeks, how often have you been bothered by the following problems?** | **Not at all** | **Several days** | **More than half the days** | **Nearly every day** |
| --- | --- | --- | --- | --- |
| a) Feeling nervous, anxious or on edge |  |  |  |  |
| b) Not being able to stop or control worrying |  |  |  |  |
| c) Worrying too much about different things |  |  |  |  |
| d) Trouble relaxing |  |  |  |  |
| e) Being so restless that it is hard to sit still |  |  |  |  |
| f) Becoming easily annoyed or irritable |  |  |  |  |
| g) Feeling afraid as if something awful might happen |  |  |  |  |

1. **In the last 3 months (since you first completed this questionnaire) have you:**

Hurt yourself on purpose **in any way** (e.g. by taking an overdose of pills, or by cutting yourself)?

Yes  No

1. **On any of the occasions (in the last three months) when you hurt yourself on purpose, did you ever seriously want to kill yourself?**

Yes  No

**Section D The following questions ask about financial stresses and strains in your life, and the help you receive.**

| **AFFORDABILITY, Please tick one box for each question** | | | | |
| --- | --- | --- | --- | --- |
| 1. **How often does it happen that you do not have enough money to:** | Often | Sometimes | Never | Don’t know |
| Afford the kinds of food that you or your family should have? |  |  |  |  |
| Afford the kinds of clothing that you or your family should have? |  |  |  |  |
| 1. **How often does it happen that you find it difficult to:** | Often | Sometimes | Never | Don’t know |
| Pay bills for gas and/or electricity? |  |  |  |  |
| Pay bills for the telephone? |  |  |  |  |
| Pay your rent or mortgage? |  |  |  |  |

| 1. **How dissatisfied or satisfied are you with the income of your household?** | | | | | | |
| --- | --- | --- | --- | --- | --- | --- |
| Completely satisfied | Mostly satisfied | Somewhat satisfied | Neither satisfied nor dissatisfied | Somewhat dissatisfied | Mostly dissatisfied | Completely dissatisfied |
|  |  |  |  |  |  |  |

| 1. **In the next 2 months how much do you anticipate that you or your family will experience financial hardships such as inadequate food, housing clothing or heating?** | | | | |
| --- | --- | --- | --- | --- |
| Never | Rarely | Sometimes | Often | Always |
|  |  |  |  |  |

| 1. **Please respond to the following statements by ticking just one box:** | **Exactly true** | **Moderately true** | **Hardly true** | **Not at all true** |
| --- | --- | --- | --- | --- |
| It is hard to stick to my spending plan when unexpected expenses arise |  |  |  |  |
| It is challenging to make progress towards my financial goals |  |  |  |  |
| When unexpected expenses occur I usually have to use credit |  |  |  |  |
| When faced with a financial challenge, I have a hard time figuring out a solution |  |  |  |  |
| I lack confidence in my ability to manage my finances |  |  |  |  |
| I worry about running out of money in retirement |  |  |  |  |

**Section E Your Work, Social & Financial Situation**

| **EMPLOYMENT**   1. **Are you currently doing paid work of any sort? [tick one]** | **Yes** | **No** |
| --- | --- | --- |
| If **Yes** (you are currently working):   **How many hours do you work per week on average?** ……………. **hours   How long have you been working in your current job?** ………….... **years** ……………**months** | | |
| If **No** (you are not currently working):  **How long have you been out of work?** ………………. **years** ………… **months**  **What is the reason you are out of work?** | | |

| **BENEFITS**   1. **Do you or your partner receive any of the following?  Please tick ALL that apply** | **You** | **Your**  **Partner** |
| --- | --- | --- |
| Job seekers allowance |  |  |
| Employment support allowance |  |  |
| Disability living allowance |  |  |
| Attendance allowance |  |  |
| Carers allowance |  |  |
| Council tax benefit |  |  |
| Housing benefit |  |  |
| Working tax credit |  |  |
| Income support |  |  |
| Statutory sick pay |  |  |
| Child benefit |  |  |
| Don’t know |  |  |
| **Other (please specify)** | | |

| **DEBT AND OTHER HARDSHIPS**   1. **Are you or your partner behind on payments for any of the following? Please tick ALL that apply** | **You** | **Your Partner** |
| --- | --- | --- |
| Mortgage |  |  |
| Rent |  |  |
| Electricity/gas/water |  |  |
| Council tax |  |  |
| Benefits (e.g. overpayments of tax credits) |  |  |
| Credit card |  |  |
| Hire Purchase (e.g. loan for care or other possessions) |  |  |
| Student loans |  |  |
| Business loans |  |  |
| Loans from family or friends |  |  |
| Short term cash loan (e.g. payday or other lenders) |  |  |
| Taxes |  |  |
| **Other (please specify)** | | |

| 1. **Which of the following organisations have you ever used in the last 3 months? Please tick ALL that apply** | | **How often?** |
| --- | --- | --- |
| General advice centres (e.g. Citizens Advice Bureau or local advice centres) |  |  |
| Housing benefit / homelessness services (e.g. Shelter) |  |  |
| Debt advice services (e.g. Talking Money and Pennywise) |  |  |
| Alcohol / drug advice services (e.g. Bristol Roads) |  |  |
| Mental health charities (e.g. MIND and Samaritans) |  |  |
| Foodbanks |  |  |
| Other advice or support for financial problems |  |  |
| **Any other (please specify)** | | |

| 1. **Which of the following NHS services have you used in the last 3 months (please tick ALL that apply):** | | **How often?** |
| --- | --- | --- |
| Primary care (e.g. GP, practice nurse) |  |  |
| Accident & Emergency visit |  |  |
| Outpatient appointment |  |  |
| Hospital admission |  |  |
| Other (please specify): |  |  |

**Section F Home and Family**

| 1. **Who is in your household?** | | **How many** |
| --- | --- | --- |
| Partner |  |  |
| Children (under 16) |  |  |
| Children (16 or over) |  |  |
| Parents |  |  |
| **Other (please specify):** | | |

| 1. **Is the accommodation in which you live:  (tick as appropriate)** | |
| --- | --- |
| Owned outright by you or your partner |  |
| Owned with mortgage/loan by you or your partner |  |
| Rented |  |
| **Other (please specify):** | |

**Contact details**

May we have a friend or family’s contact details in case we are unable to get hold of you?

Address

Telephone

Email

**MANY THANKS FOR ANSWERING THESE QUESTIONS**
