# Supplementary material for: Preventing male suicide through a psychosocial intervention that provides psychological support and tackles financial difficulties: a mixed method evaluation
Source: BMC Psychiatry. 2022 May 13;22:333. doi: 10.1186/s12888-022-03973-5 (PMC9103598; doi:10.1186/s12888-022-03973-5)
Supplement: Supplementary file 2 — Additional file 2. [file 12888_2022_3973_MOESM2_ESM.docx]

**Hope Service User Topic Guide Questions**

1. We would like to get some background information on who we’re speaking with, if you’re happy to provide us with these details.
   1. Could you share with us your age range e.g. forties, fifties.
   2. How would you describe your ethnic background?
2. When did you last have a session with the Hope service?
3. Do you remember how many sessions you had with a Hope worker?
   1. Did you always see the same person at HOPE sessions or did you see different people?
   2. Did you have the sessions face to face or over the phone? What was that like? What was the difference like between them?
   3. If you had face to face sessions where did you meet?
   4. What was it like talking to the Hope worker?
   5. Did you feel able to say everything that you wanted to?
   6. Can you talk me through what happens in a Hope session?
   7. Did you get what you needed out of the sessions?
   8. Could you tell me how the relationship with the Hope worker has developed over time / sessions?
4. Did you have any other form of contact other than sessions? i.e. phone conversations or texts messages?
   1. Were these helpful in between sessions? How?
5. Can you tell me briefly about your situation that led you to come to the Hope service?
   1. How did you make the connection with HOPE?
6. Did you get enough sessions and long enough sessions with Hope?
7. How did Hope help you manage the issues that you were facing? How have these issues changed since accessing the Hope service?
   1. (Extra prompt if needed?) How has the Hope service made a difference to you?
8. Did you get referred to other organisations by the Hope service? Did you go to those organisations and did they help?
   1. How does the Hope service compare to other forms of support you have accessed?
9. What’s been the impact of the HOPE sessions for you/on your life?
   1. How is your general financial stability/employment situation now?

**COVID-19**

1. Have you had any Hope sessions since the coronavirus lockdown? (If yes, ask a and b)
   1. Has the HOPE service been able to support you during the lockdown? How has this worked out for you?
   2. Has the form of support been different during the pandemic (e.g. online or telephone rather than face to face)? What was that like?
2. Is there anything else that the HOPE service could do to support you either during the coronavirus lockdown or more generally, if they were able to?
3. Would you have any other suggestions on how the HOPE service could be improved?
4. Is there anything else that you would like to add?
5. Do you have any comments or feedback on the questions we have asked in this interview?
