# Supplementary material for: Preventing male suicide through a psychosocial intervention that provides psychological support and tackles financial difficulties: a mixed method evaluation
Source: BMC Psychiatry. 2022 May 13;22:333. doi: 10.1186/s12888-022-03973-5 (PMC9103598; doi:10.1186/s12888-022-03973-5)
Supplement: Supplementary file 3 — Additional file 3. [file 12888_2022_3973_MOESM3_ESM.docx]

| Characteristics of service users who completed the questionnaire at baseline, 6-months and at baseline only | | | | | | | |
| --- | --- | --- | --- | --- | --- | --- | --- |
|  | | **All baseline** | | **Baseline and follow-up** | | **Baseline only** | |
| **Variable** | | **(n=105)*** | | **(n=80)**** | | **(n=25)***** | |
|  |  | **n/mean(SD)** | **%/range** | **n/mean(SD)** | **%/range** | **n/mean(SD)** | **%/range** |
| Age | | 47.4 (8.8) | 29.3-64.0 | 47.3 (8.5) | 30.9-64.0 | 47.5 (10.0) | 29.3-64.0 |
| Ethnicity: | |  |  |  |  |  |  |
|  | White | 87 | 82.9 | 66 | 82.5 | 21 | 84.0 |
|  | BAME | 15 | 14.3 | 13 | 16.3 | 2 | 8.0 |
|  | Unknown | 3 | 2.9 | 1 | 1.3 | 2 | 8.0 |
| In employment | |  |  |  |  |  |  |
|  | No | 80 | 76.2 | 64 | 80.0 | 16 | 64.0 |
|  | Yes | 20 | 19.0 | 15 | 18.8 | 5 | 20.0 |
|  | Unknown | 5 | 4.8 | 1 | 1.3 | 4 | 16.0 |
| Depression severity | | 19.8 (5.3) | 5.0-27.0 | 20.2 (5.5) | 5.0-27.0 | 18.5 (4.6) | 12.0-27.0 |
| Depression category: | |  |  |  |  |  |  |
|  | mild (5-9) | 4 | 3.8 | 4 | 5.0 | 5 | 20.0 |
|  | moderate (10-14) | 16 | 15.4 | 11 | 13.8 | 8 | 32.0 |
|  | moderately severe (15-19) | 22 | 21.2 | 14 | 17.5 | 11 | 44.0 |
|  | severe (20-27) | 62 | 59.6 | 51 | 63.8 | 1 | 4.0 |
| Financial self-efficacy | | 10.8 (4.3) | 6.0-24.0 | 11.1 (4.6) | 6.0-24.0 | 10.0 (3.4) | 6.0-16.0 |
| Suicidal ideation | |  |  |  |  |  |  |
|  | No | 3 | 2.9 | 3 | 3.8 | 0 | 0.0 |
|  | Yes | 102 | 97.1 | 77 | 96.3 | 25 | 100.0 |
| Suicide attempt | |  |  |  |  |  |  |
|  | No | 50 | 47.6 | 43 | 53.8 | 7 | 28.0 |
|  | Yes | 55 | 52.4 | 37 | 46.3 | 18 | 72.0 |
| No. of benefits claimed: | |  |  |  |  |  |  |
|  | 0 | 34 | 32.4 | 24 | 30.0 | 10 | 40.0 |
|  | 1-2 | 47 | 44.8 | 36 | 45.0 | 11 | 44.0 |
|  | 3-4 | 23 | 21.9 | 19 | 23.8 | 4 | 16.0 |
|  | 5+ | 1 | 1.0 | 1 | 1.3 | 0 | 0.0 |
| Financial hardships (no. of payments behind on): | |  |  |  |  |  |  |
|  | 0 | 50 | 47.6 | 36 | 45.0 | 14 | 56.0 |
|  | 1-2 | 32 | 30.5 | 26 | 32.5 | 6 | 24.0 |
|  | 3-4 | 17 | 16.2 | 13 | 16.3 | 4 | 16.0 |
|  | 5+ | 6 | 5.7 | 5 | 6.3 | 1 | 4.0 |
